# Coexistence of superficial carcinogenesis of resident epithelium besides neuroendocrine neoplasm of the digestive tract

**DOI:** 10.1002/cam4.4485

**Published:** 2022-01-20

**Authors:** Hiroyuki Kuwano, Takehiko Yokobori, Munenori Ide, Hiroshi Saeki, Makoto Sohda, Makoto Sakai, Tomonori Yoshida, Kengo Kuriyama, Kyoichi Ogata, Hiroomi Ogawa, Takuhisa Okada, Tatsuya Miyazaki, Shunsuke Takahashi, Ken Shirabe

**Affiliations:** ^1^ Department of General Surgical Science Gunma University Graduate School of Medicine Maebashi Japan; ^2^ Fukuoka City Hospital Fukuoka Japan; ^3^ Division of Integrated Oncology Research Gunma University Initiative for Advanced Research (GIAR) Maebashi Japan; ^4^ Department of Pathology Maebashi Red Cross Hospital Maebashi Japan; ^5^ Department of Surgery Maebashi Red Cross Hospital Maebashi Japan

**Keywords:** bystander effect, MiNEN, NEC, NET, neuroendocrine carcinoma

## Abstract

**Background & Aims:**

Mixed neuroendocrine–non‐neuroendocrine neoplasm (MiNEN) is a rare neuroendocrine neoplasm (NEN) comprising dual neuroendocrine and non‐neuroendocrine components. Although the coexistence pattern of neuroendocrine and non‐neuroendocrine components in definitive MiNEN is thought to overlap, there may be a coexistent pattern of both components, such as superficial carcinoma adjacent to NEN. The present study evaluated the histopathological findings of the coexistence pattern of superficial carcinomas adjacent to NENs in the esophagogastrointestinal tract.

**Methods:**

From 2000 to 2019, 35 serial NEN resections of the esophagus (*n* = 9), stomach (*n* = 3), and large intestine (*n* = 23), respectively, were performed at Gunma University Hospital. Borderline areas between NEN and resident superficial epithelium were observed in the 35 serial NEN cases as well as two additional cases from affiliated hospitals.

**Results:**

Among the 35 serial NEN samples, squamous cell carcinomatous/dysplastic components were identified 77.8% (7/9 cases) of esophageal NENs, and adenocarcinomatous areas were seen in 66.7% (2/3 cases) of gastric NENs and 26% (6/23 cases) of colorectal NENs. Thus, all superficial carcinomatous components adjacent to NENs were observed as squamous cell carcinoma/dysplasia in esophagus and adenocarcinoma in stomach and large intestine, which showed histological characteristics as the resident epithelial pattern in each organ.

**Conclusions:**

These findings suggested a potential “paratransformation” or “bystander effect” in resident epithelium by NENs. Thus, “bystander carcinogenesis” could be a pathogenic mechanism of resident epithelium transformation adjacent to NENs in the esophagogastrointestinal tract.

## Graphical abstract

All superficial carcinomatous components adjacent to neuroendocrine neoplasms were observed as squamous cell carcinoma/dysplasia in esophagus and adenocarcinoma in stomach and large intestine, which showed histological characteristics as the resident epithelial pattern in each organ. Therefore, bystander carcinogenesis could be a pathogenic mechanism of resident epithelium transformation adjacent to neuroendocrine neoplasms in the esophagogastrointestinal tract.

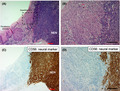

**SUMMARY**

Superficial carcinomas adjacent to neuroendocrine neoplasms were squamous cell carcinoma/dysplasia in the esophagus and adenocarcinoma in the stomach/large intestine. Bystander carcinogenesis could be a pathogenic mechanism of resident epithelium transformation adjacent to esophagogastrointestinal neuroendocrine neoplasms.

## 1 INTRODUCTION

Mixed neuroendocrine–non‐neuroendocrine neoplasms (MiNENs) were previously considered to be a rare neuroendocrine tumor (NET); however, there have been several reports of this tumor occurring in digestive tract.^1^, ^2^ MiNENs are composed both of neuroendocrine and non‐neuroendocrine components. The combination of neuroendocrine and non‐neuroendocrine components ranges widely and includes pure neuroendocrine neoplasms, neoplasms with a focal non‐neuroendocrine component, non‐neuroendocrine with interspersed neuroendocrine cells, and pure non‐neuroendocrine neoplasms. MiNENs are defined as neoplasms in which each component represents 30% of the whole tumor.^3^, ^4^ However, although some tumors may comprise a combination of non‐neuroendocrine and neuroendocrine components, MiNENs diagnosed strictly according to this definition are really found. In some tumors composed of neuroendocrine and non‐neuroendocrine components, both components occur in separate areas within the same lesion and are thought to be collision or composite tumors. Another pattern of this tumor type shows diffusely or focally intermingled admixed features of both components. Moreover, non‐neuroendocrine and neuroendocrine characteristics are present in the same neoplastic cells in amphicrine tumors.^5^ La Rosa et al. reported the histopathological features of a case of anorectal neuroendocrine neoplasm (NEN) that showed focal squamous cell differentiation, as well as another case of adenocarcinoma and scattered neuroendocrine cells detected using anti‐chromogranin A antibody, and showed that the cases could not be classified as MiNEN according to the definition.^2^

The histopathological definition of NEN is important for the diagnosis, analyses of its malignancy potential, and therapeutic strategy. However, the patterns of coexistence of the non‐neuroendocrine and neuroendocrine components are also important to determine the histogenesis of NENs with two components.

The present study analyzed the results of serial retrospective histopathological investigations of resected specimens of NEN of the esophagogastrointestinal tract. The histogenesis of the pattern of the tumors was examined, considering the possibility of “paratransformation” or “bystander carcinogenesis” in resident epithelium with NEN coexistence.

## 2 METHODS

### 2.1 Patient information

The present study analyzed surgically resected specimens from 35 serial NEN patients at Gunma University Hospital and two additional NEN patients at affiliated hospitals from 2000 to 2019. A total of 37 cases were pathologically diagnosed as NEN and included in this study. Among these, 10 patients had esophageal NEN, four had gastric NEN, and 23 had colorectal NEN. Superficial carcinomatous/dysplastic areas coexisting adjacent to NEN were identified retrospectively, and the clinicopathological characteristics were investigated and immunohistochemical staining was performed to detect CD56 and synaptophysin as neural markers. Histopathological classification of NEN was performed according to the 2019 World Health Organization classification of tumors of the digestive system.^6^ Clinical samples were used in accordance with the institutional guidelines and the Helsinki Declaration (approval number: HS2020‐138). Patient agreement was obtained using the opt‐out method.

### 2.2 Immunohistochemical analysis

Immunohistochemical staining was performed on 4‐mm sections consecutively cut using a Ventana Benchmark Ultra according to the manufacturer's instructions. For the primary antibody reaction, sections were incubated with CD56 rabbit monoclonal antibody (MRQ‐42) (Nichirei) or synaptophysin rabbit monoclonal antibody (SP11) (Roche Diagnostics). CD56 and synaptophysin expression was evaluated as representative neural markers for NEN to distinguish the NEN area from non‐neuroendocrine components.

### 2.3 Patient and public involvement

Patients and the public were not involved in the design and execution of this study.

## 3 RESULTS

### 3.1 Coexistence of superficial carcinoma adjacent to NENs in the digestive tract

The present study analyzed resected sections from 37 NEN patients, including serial 35 patients treated at Gunma University Hospital and two patients treated at affiliated hospitals, and evaluated the coexistence of superficial carcinoma adjacent to NENs. The 35 serial cases at Gunma University were selected to evaluate the incidence rate of coexistence of superficial carcinomatous/dysplastic areas adjacent to the NEN components.

**FIGURE 1:**
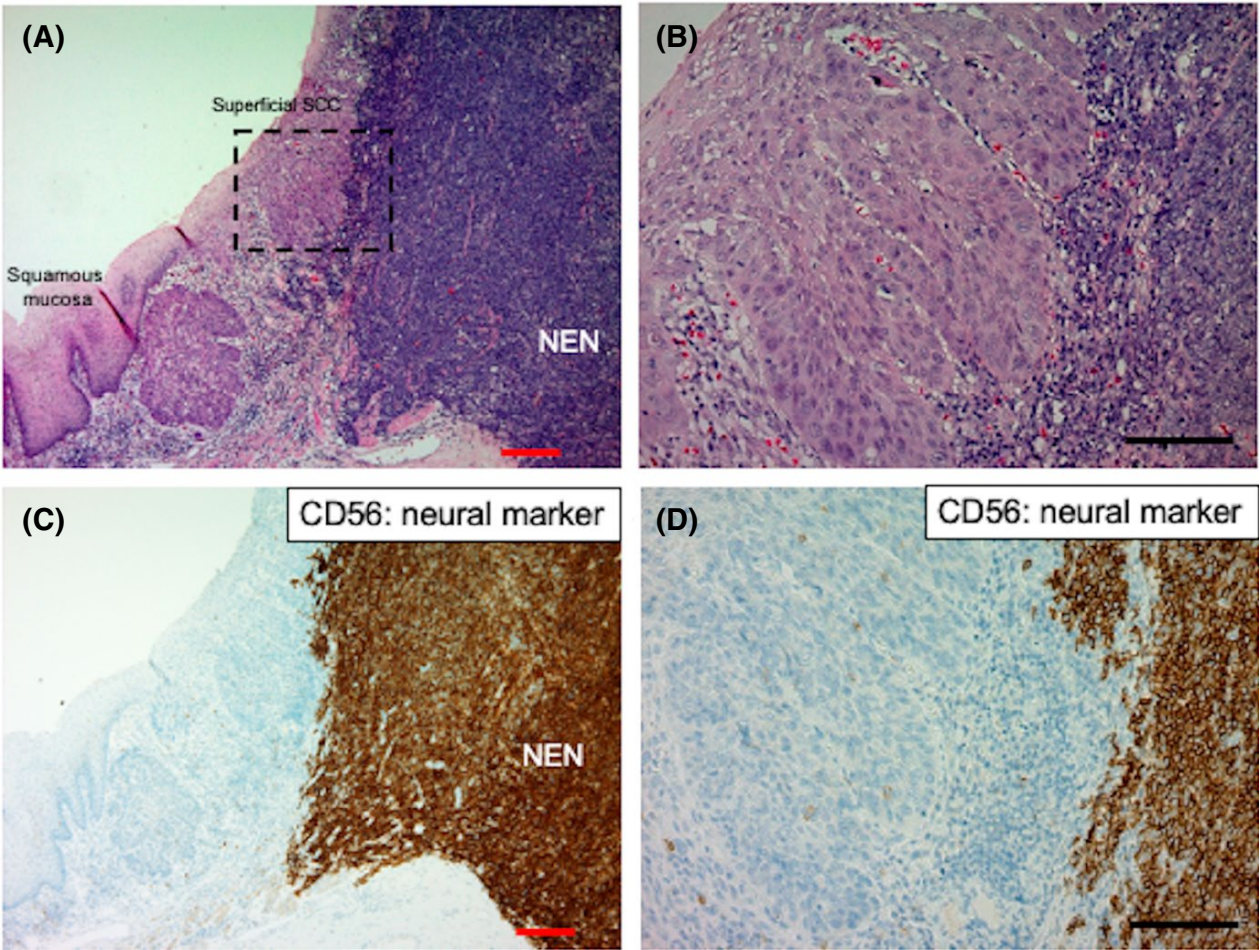
Histological distribution of NEN and adjacent SCC in a representative esophageal NEN sample. (A) Low‐ and (B) high‐power view of the tumor distribution of NEN and adjacent superficial SCC in a resected esophageal NEN sample. (C) Low‐ and (D) high‐power view of neural marker CD56 expression pattern of NEN and adjacent superficial SCC in a resected esophageal NEN sample. Red and black bars indicate 200 μm and 100 μm, respectively. NEN, neuroendocrine neoplasm; SCC, squamous cell carcinoma

Among nine cases of serial esophageal NENs, all underwent esophagectomy (Table 1). Among these, squamous cell carcinoma (SCC) components were identified in six (66.7%) esophageal NENs. Including one case of coexistence of squamous cell dysplasia, 77.8% (7/9) of serial esophageal NENs were accompanied by superficial squamous epithelial carcinomatous/dysplastic lesions (Table 1). Representative histopathological features using neural markers and squamous cell marker p63 are shown in Figure 1 and Figure S1.

**TABLE 1:** Clinicopathologic characteristics of neuroendocrine neoplasm (esophagus)

Case	Age/sex	Treatment	NEN					Adjacent coexistence of superficial carcinomatous area
			Diagnosis	pT	pN	M	pStage	
1	67/M	Adjuvant chemotherapy, esophagectomy	Endocrine cell carcinoma	3	2	0	III	SCC
2	72/M	Esophagectomy	Endocrine cell carcinoma	2	1	0	III	None
3	59/M	Esophagectomy	Endocrine cell carcinoma	3	1	0	III	SCC
4	66/F	Esophagectomy	Small‐cell neuroendocrine carcinoma	1	1	0	II	None
5	76/M	Esophagectomy	Small‐cell neuroendocrine carcinoma	1	0	0	I	Intraepithelial squamous dysplasia
6	57/M	Esophagectomy	Small‐cell neuroendocrine carcinoma	1	0	0	I	SCC
7	63/F	Esophagectomy	Small‐cell neuroendocrine carcinoma	1	0	0	I	SCC
8	70/M	Esophagectomy	Small‐cell neuroendocrine carcinoma	1	0	0	I	SCC
9	80/M	Esophagectomy	Large‐cell neuroendocrine carcinoma	3	3	0	III	SCC
10^a^	51/M	Esophagectomy	Large‐cell neuroendocrine carcinoma	1	1	0	II	Adenocarcinoma/dysplastic change of resident ducts. Atypia of resident grands and ducts.

Abbreviations: NEN, neuroendocrine neoplasm; SCC, squamous cell carcinoma.

^a^ NEN case in an affiliation hospital.

**FIGURE 2:**
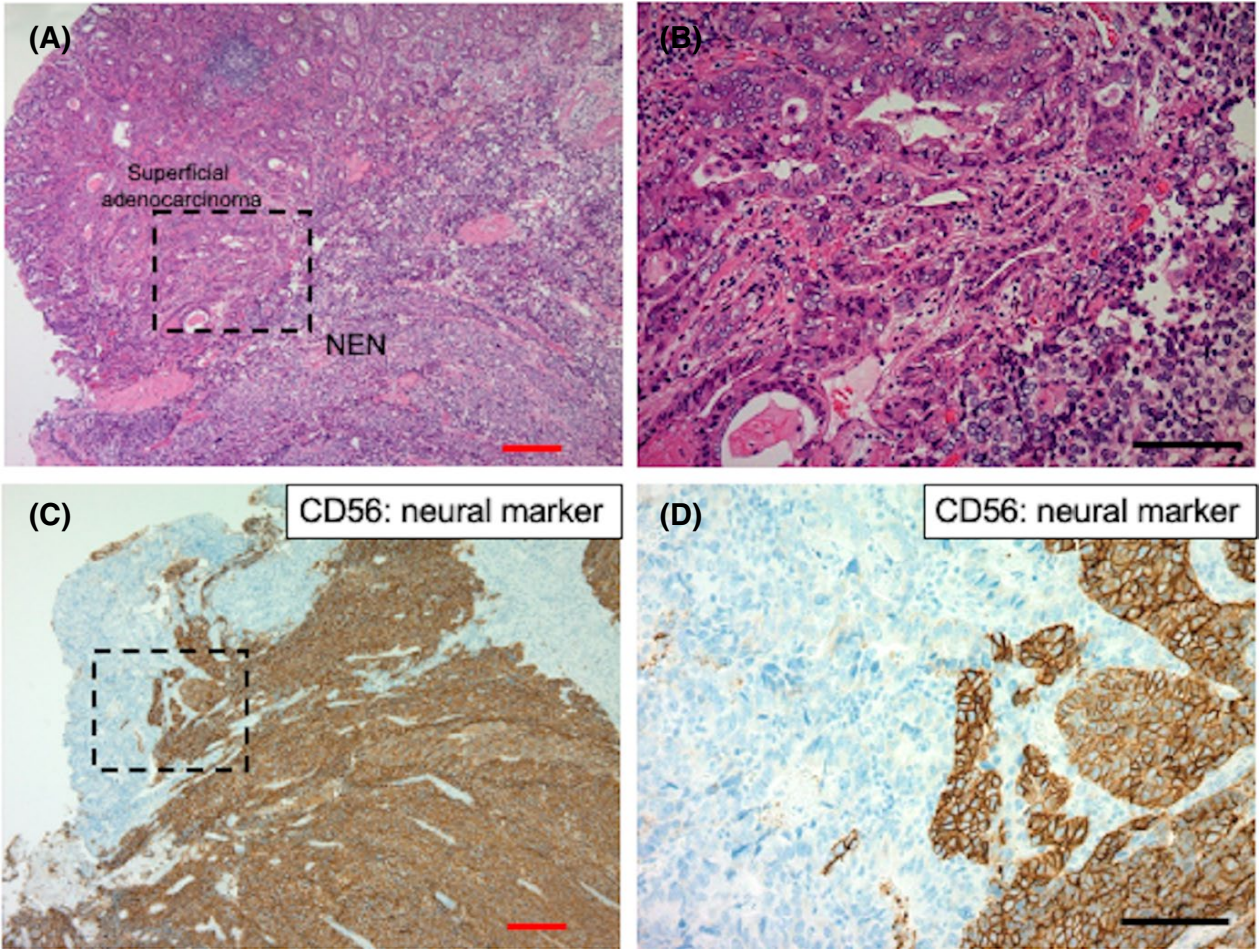
Histological distribution of NEN and adjacent gastric adenocarcinoma in a representative gastric NEN sample. (A) Low‐ and (B) high‐power view of tumor distribution of NEN and adjacent superficial gastric adenocarcinoma in a resected gastric NEN sample. (C) Low‐ and (D) high‐power view of neural marker CD56 expression pattern of NEN and adjacent gastric adenocarcinoma in a resected gastric NEN sample. Red and black bars indicate 200 μm and 100 μm, respectively. NEN, neuroendocrine neoplasm

Among three gastric NENs, superficial adenocarcinomas coexisted in the specimens from the two cases of NET G3 and large‐cell endocrine carcinoma. Thus, adenocarcinomatous areas were seen in two (66.7%) cases of serial gastric NENs (Table 2). Representative histopathological features of coexisting superficial gastric adenocarcinoma using neural markers are shown in Figure 2 and Figures S2 and S3.

**TABLE 2:** Clinicopathologic characteristics of neuroendocrine neoplasm (stomach)

Case	Age/sex	Treatment	NEN					Adjacent coexistence of superficial carcinomatous area
			Diagnosis	pT	pN	M	pStage	
1	49/F	Gastrectomy	NET (G2)	2	0	0	II	None
2	84/M	Gastrectomy	NET (G3)	3	2	0	III	Adenocarcinoma
3	74/M	Gastrectomy	Large‐cell endocrine carcinoma	2	0	0	I	Adenocarcinoma
4^a^	73/M	Gastrectomy	NET (G3)	1	3	1	IV	Adenocarcinoma

Abbreviations: NEN, neuroendocrine neoplasm; NET, neuroendocrine tumor.

^a^ NEN case in an affiliation hospital.

**FIGURE 3:**
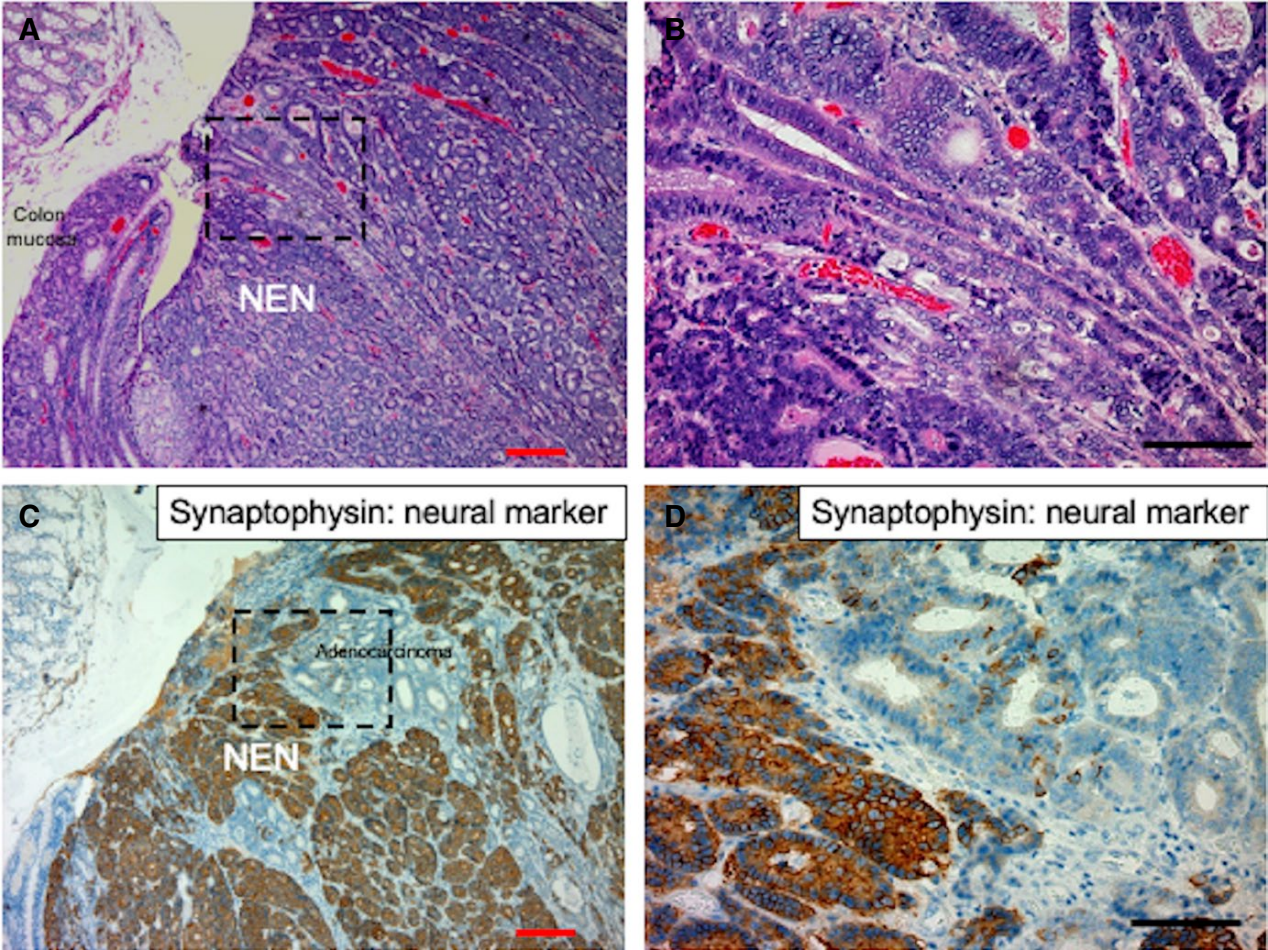
Histological distribution of NEN and adjacent colon adenocarcinoma in a representative colon NEN sample. (A) Low‐ and (B) high‐power view of the tumor distribution of NEN and colon adenocarcinoma in a resected colon NEN sample (case 1 in Table 3). (C) Low‐ and (D) high‐power view of the neural marker synaptophysin expression pattern of NEN and adjacent colon adenocarcinoma in a resected colon NEN sample. Red and black bars indicate 200 μm and 100 μm, respectively. NEN, neuroendocrine neoplasm

Colon adenocarcinomatous components adjacent to large‐cell neuroendocrine carcinoma coexisted superficially in all three cases (100%) of colonic NENs (Table 3). Among 20 cases of serial rectal NENs, endoscopic mucosal resection was performed in two cases, transanal resection in 12 cases, and surgical rectal resection in six cases. Rectal adenocarcinomatous components were identified in three cases (15%) of 20 serial rectal NENs (Table 3). The frequency of coexistence of superficial carcinomatous components was higher in cases with colon large‐cell neuroendocrine carcinoma (100%) than rectal NET and MiNEN (15%). Additionally, three cases with rectal NENs showed adenocarcinomas components distant from the NEN components that were not counted as adjacent coexistence of superficial carcinomatous area in the present study (Table 3). Representative histopathological features of coexisting superficial colon adenocarcinoma are shown in Figure 3.

**TABLE 3:** Clinicopathological characteristics of neuroendocrine neoplasm (colon and rectum)

Case	Age/sex	Location	Treatment	NEN					Adjacent coexistence of superficial carcinomatous area
				Diagnosis	pT	pN	M	pStage	
1	62/F	Ascending colon	Colectomy	Large‐cell neuroendocrine carcinoma	4	2	1	IV	Adenocarcinoma
2	69/F	Transverse colon	Colectomy	Large‐cell neuroendocrine carcinoma	4	2	1	IV	Adenocarcinoma
3	62/M	Sigmoid colon	Colectomy	Large‐cell neuroendocrine carcinoma	4	1	1	IV	Adenocarcinoma
4	55/F	Rectum	Endoscopic mucosal resection	NET (G1)	1	X	0	I	None
5	77/M	Rectum	Endoscopic mucosal resection	NET (G2)	1	X	0	I	None
6	38/M	Rectum	Transanal resection	NET (G1)	1	X	0	I	None
7	50/F	Rectum	Transanal resection	NET (G1)	1	X	0	I	None
8	52/M	Rectum	Transanal resection	NET (G1)	1	X	0	I	None
9	60/M	Rectum	Transanal resection	NET (G1)	1	X	0	I	None
10	63/F	Rectum	Transanal resection	NET (G1)	1	X	0	I	None
11	66/M	Rectum	Transanal resection	NET (G1)	1	X	0	I	None
12	77/F	Rectum	Transanal resection	NET (G1)	1	X	0	I	None
13	82/M	Rectum	Transanal resection	NET (G1)	1	X	0	I	None
14	41/M	Rectum	Transanal resection	NET (G1)	1	X	0	I	Adenocarcinoma
15	43/M	Rectum	Transanal resection	NET (G1)	1	X	0	I	Adenocarcinoma
16	53/F	Rectum	Transanal resection	NET (G1)	1	X	0	I	Adenocarcinoma
17	54/M	Rectum	Transanal resection	NET (G1)	1	X	1	IV	None: Adenocarcinoma (a little away)
18	74/M	Rectum	Low anterior resection	NET (G1)	2	1	0	III	None
19	72/M	Rectum	Low anterior resection	NET (G1)	2	1	0	III	None: Adenocarcinoma in adenoma (a little away)
20	44/F	Rectum	Low anterior resection	NET (G2)	1	1	0	III	None: adenocarcinoma (a little away)
21	57/M	Rectum	Low anterior resection	NET (G2)	2	0	0	I	None
22	43/F	Rectum	Low anterior resection	NET (G2)	3	1	1	IV	None
23	80/M	Rectum	Hartmann's operation	MiNEN	3	1	1	IV	None

Abbreviations: MiNEN, mixed neuroendocrine–non‐neuroendocrine neoplasm; NEN, neuroendocrine neoplasm; NET, neuroendocrine tumor.

### 3.2 Carcinomatous/dysplastic transformation of resident epithelial tissues in the esophageal NEN

All superficial carcinomas, including six esophageal SCC, two gastric adenocarcinomas, and six colorectal adenocarcinomas were located adjacent to NEN components and resident anatomical constructions including esophageal squamous epithelium, gastric mucosa, and colorectal mucosa in the present study. Thus, carcinomatous components were all SCCs in the esophagus and adenocarcinoma in the stomach and large intestine, of which histological characteristics were the resident original epithelial pattern in each organ.

**FIGURE 4:**
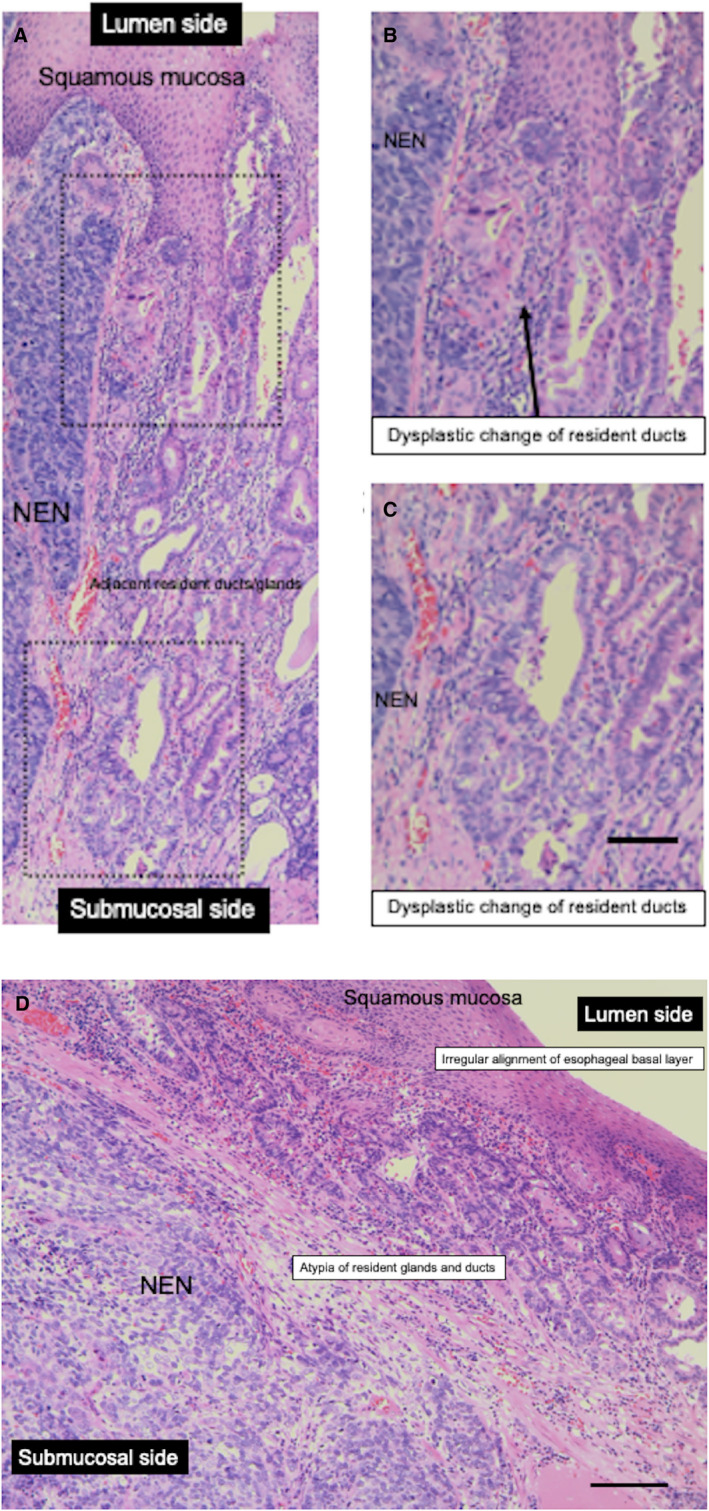
Histological transformation of resident ducts/glands in a representative esophageal NEN sample. (A) NEN‐adjacent dysplasia of ducts in a resected esophageal NEN sample (in the esophagogastric junction, the NEN tumor invades submucosa) (case 10 in Table 1). (B), (C) High‐power view of NEN‐adjacent transformation of resident ducts in a resected esophageal NEN sample. (D) NEN‐adjacent atypia of resident glands and ducts in a resected esophageal NEN sample. Black bar indicates 100 μm. NEN, neuroendocrine neoplasm

We focused on one case with esophageal NEN (Figure 4). The dysplastic component in the resident ducts was observed adjacent to the NEN components (Figure 4A–C). The resident glands and ducts adjacent to NEN components were also transformed (Figure 4D). Such histological atypia was more prominent in resident glands and ducts of the esophagus, which were located closer to NENs than squamous epithelium.

## 4 DISCUSSION

Cells communicate with their immediate neighbors via adhesion molecules or tunneling nanotubules.^7^, ^8^, ^9^ Communication with nearby or distant cells can be achieved by soluble factors, such as cytokines, chemokines, and nanovesicles.^10^ The term “bystander effect” describes a type of cell‐to‐cell interaction^11^ in which cells that have not been directly exposed irradiation are altered to exhibit damaging effects by stress signals from nearby irradiated cells. This phenomenon has been extensively studied not only in the field of irradiation treatment^12^, ^13^, ^14^ but also in cancer chemotherapy^15^ and suicide gene therapy.^16^, ^17^, ^18^

Cell‐to‐cell interaction is not only recognized in cancer treatment effects, but also in therapeutic resistance. We recently demonstrated that exosomal microRNAs derived from gefitinib‐resistant lung cancer cells induced drug resistance in a sensitive cell line.^19^ Thus, cell‐to‐cell communication or interactions including a “bystander effect” are a possible biological phenomenon in nature.

We investigated the mechanisms of carcinogenesis and mode of progression of cancer tissues, focusing on the multicentric or “field carcinogenesis” in esophageal cancer^20^, ^21^, ^22^, ^23^ in contrast to the concept of single‐cell origin of cancer tissue. Field carcinogenesis would include the possibility of multifocal and/or multi‐cellular carcinogenesis. In the early manifestation of carcinogenesis, such as in‐situ SCC, the proliferative activities of the front outer edge and center of such intraepithelial carcinoma were similar. On the other hand, cell proliferation activity of the front area was higher than that of the central area of the invasive site of cancer tissue, suggesting that proliferation is the main mechanism in invasive and metastatic sites, and that early events of such carcinogenesis would include cell‐to‐cell interaction of carcinogenesis, which is interpreted as malignant transformation of bystander non‐cancerous cells influenced by an initial transformed single cell or several cells.^24^ We proposed “paratransformation” as a potential model of origin for esophageal SCC.^25^ Moreover, we used an animal experiment to demonstrate that KatoIII and Lu135 derived from human cancer tissue showed the potential for atypical or malignant transformation in the adjacent anorectal epithelium of tumor‐inoculated mice,^26^ which could demonstrate the concept of cell‐to‐cell interaction or “bystander effect” in carcinogenesis.

In the present histopathological investigation of superficial carcinoma located adjacent to NENs of the digestive tract, we considered the histogenesis of such carcinoma. According to the concept of single‐cell origin of this type of tumor tissue, pluripotential cancer stem cells proliferate and differentiate toward both carcinomatous and neuroendocrine tumors, or carcinoma or NENs partially transformed to another cell type. On the other hand, another possibility for the histogenesis of such tumor is the “bystander effect” of carcinogenesis. Namely, NENs possess the potential for “bystander effect” or “paratransformation” of epithelial tissues located adjacent to NENs. This concept is supported as follows. First, almost all superficial carcinomatous lesions in the present study were noninvasive and carcinoma‐in‐situ status or those with focal invasion sites located superficially adjacent to NENs. Such findings are likely due to different mechanisms of carcinogenesis of each component, rather than bidirectional histological differentiation after single pluripotential cancer stem cell.

Second, histopathological types of carcinomas in this series depend on the resident original epithelial characteristics, such as SCC occurring in the esophagus and adenocarcinoma in the stomach and colorectum.

Third, both components of the superficial carcinomas and NENs are clearly located in the style of a collision tumor rather than intricately intermingled with NENs and carcinomatous components. Furthermore, one case demonstrated esophageal NEN with histological atypia that originated from resident ducts/glands mainly located in the sub‐epithelial layer of the esophagus (Figure 4).

In terms of the malignant potential of NENs with superficial carcinomas in the esophagus and stomach, all NENs were neuroendocrine carcinoma and NET (G3). Coexistence of superficial carcinoma was detected in all patients with colon neuroendocrine carcinomas.

This study detected the coexistence of resident epithelial carcinoma and NEN in the digestive tract, and the almost resident superficial carcinoma components were adjacent to the NEN components. Therefore, we proposed that the bystander effect or paratransformation of the resident epithelium was influenced by NEN components. However, our hypothesis might not explain why resident epithelial tumors adjacent to NEN were superficial carcinoma. For instance, the coexistence of NET (G1) tumors without proliferation potency and aggressive epithelial carcinoma is expected to cause tumors with a large carcinoma component and small NET (G1) component; however, we did not observe this phenomenon in our serial NEN cohort. Therefore, we had the following discussions. First, pathological diagnosis might overlook small NET (G1) components in advanced adenocarcinoma components. Second, the resected area of background colorectal mucosa might influence a diagnosis of the coexistence of NEN and non‐NEN components. This study did not detect an adenocarcinoma component adjacent to rectal NET (G1) tumors that had been resected by endoscopic mucosal resection. However, we observed adenocarcinoma components adjacent to and slightly spaced apart from rectal NET (G1) tumors that had been resected by transanal resection or low anterior resection with a resection margin of background mucosa.

The slightly spaced apart adenocarcinoma might have originated in the background rectal mucosa, which would mean that the adenocarcinoma was not caused by bystander carcinogenesis or paratransformation, as our hypothesis suggested. When presenting the data for serial NEN cases, this study did not exclude adenocarcinoma that was slightly spaced apart from rectal NET (G1) tumors without high proliferation potency. Third, the degree of intensity of the paratransformation signal from NEN might depend on the distance between the transformed‐resident epithelium and NENs rather than the malignant/functional potential of NENs. In other words, proliferative stimulation against the transformed‐resident epithelium might be limited when the carcinoma is adjacent to the NENs in a manner that impacts paracrine signaling from NENs to the resident epithelium. Future research is necessary to evaluate the relationship between resident epithelial carcinogenesis and colorectal NET tumors based on the NET classification and distance between NEN and non‐NEN components.

The present study has some limitations, such as a lack of genetic analyses of histological components as well as a lack of investigations of potential factors such as cytokines, exosomes, and microRNAs influencing cell‐to‐cell interactions in bystander carcinogenesis. Genetic analyses of both histological components are required to elucidate the molecular mechanisms and prove our hypothesis regarding bystander carcinogenesis of the resident epithelium by adjacent NENs.

In conclusion, the present histopathological serial investigation demonstrated that the coexistence of superficial carcinomas located adjacent to NENs in the digestive tract was not rare. We propose “bystander carcinogenesis” by NENs as potential pathogenic mechanisms of resident epithelium transformation adjacent to NENs in the esophagogastrointestinal tract.

## CONFLICT OF INTEREST

The authors have no conflict of interest to disclose.

## AUTHOR CONTRIBUTION

HK and TY were involved in study concept and design. HK, MI, HS, MS, MS, TY, KK, KO, HO, TO, TM, and ST were involved in acquisition of data, and analysis and interpretation of data. HK, TY, MI, and HS were involved in drafting of the manuscript. HK, TY, and KS were involved in critical revision of the manuscript for important intellectual content.

## ETHICS STATEMENT

This study was performed in accordance with the institutional guidelines and the Helsinki Declaration (approval number: HS2020‐138).

## Supporting information

FigS1

FigS2

FigS3

## Data Availability

The data that support the findings of this study are available from the corresponding authors upon reasonable request.
