# Supplementary figures and images for: Coexistence of superficial carcinogenesis of resident epithelium besides neuroendocrine neoplasm of the digestive tract
Source: Cancer Med. 2022 Jan 20;11(4):983–92. doi: 10.1002/cam4.4485 (PMC8855898; doi:10.1002/cam4.4485)

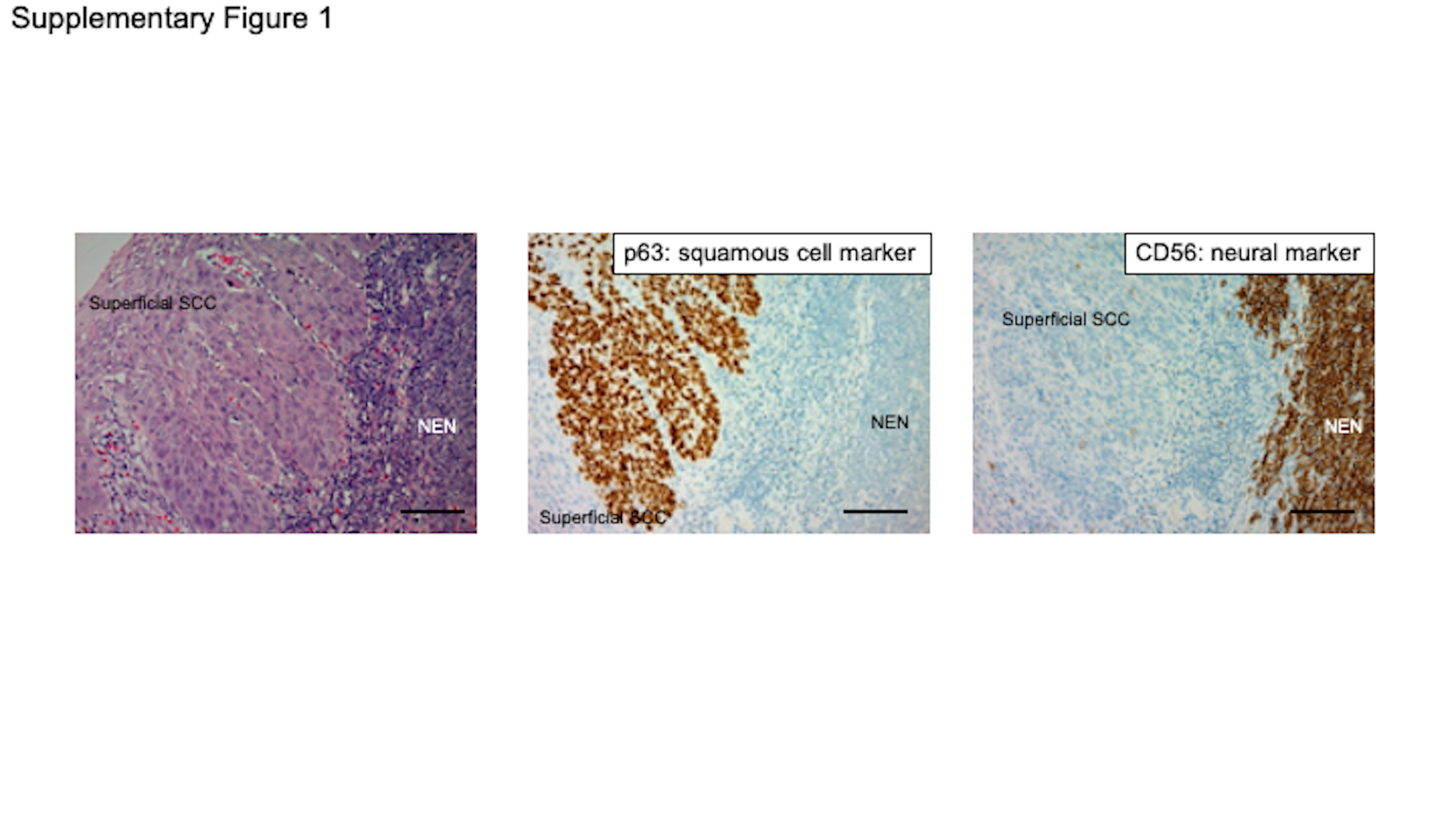

Supplement: Supplementary file 1 — FigS1 [file CAM4-11-983-s002.png]

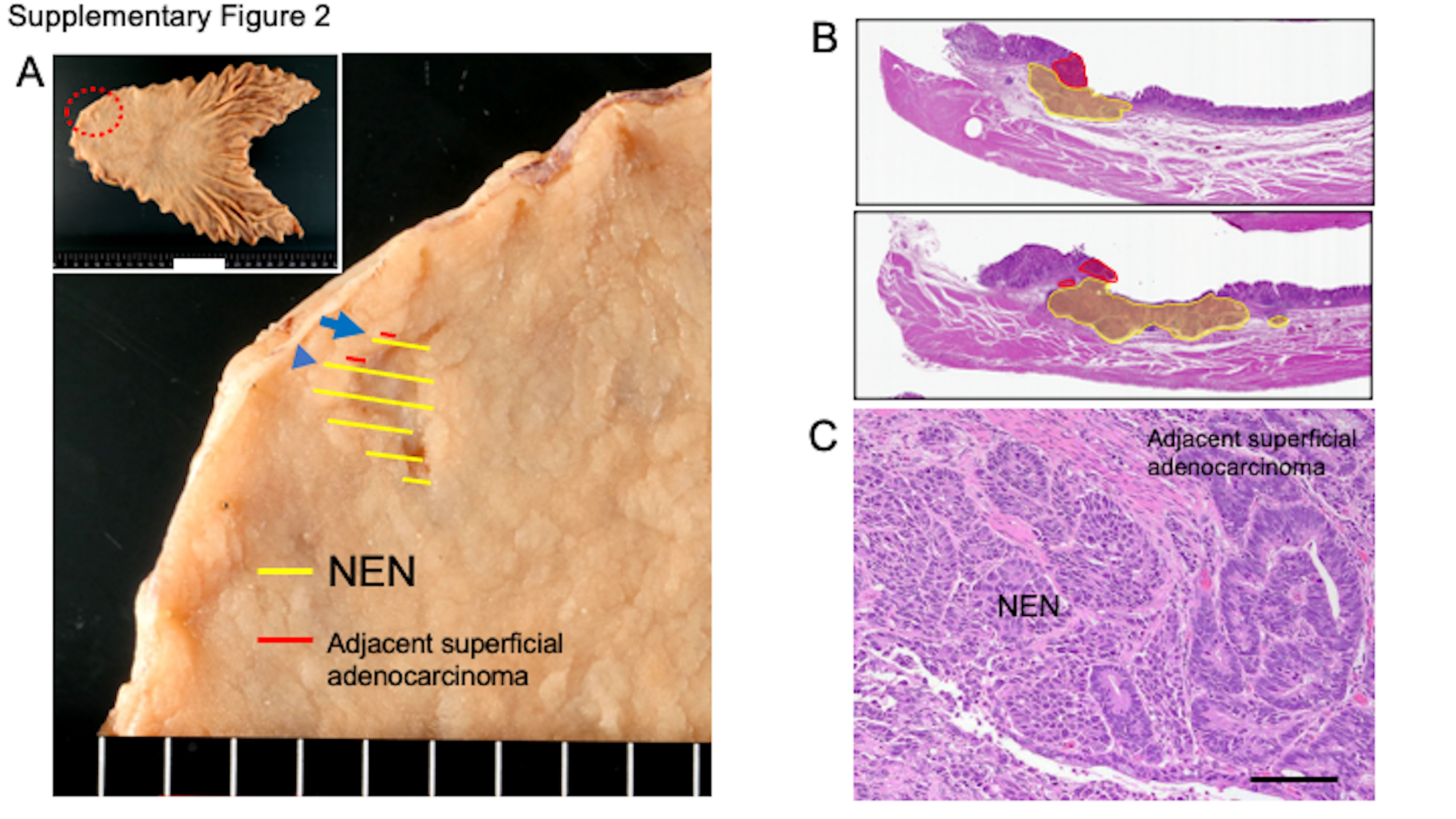

Supplement: Supplementary file 2 — FigS2 [file CAM4-11-983-s003.png]

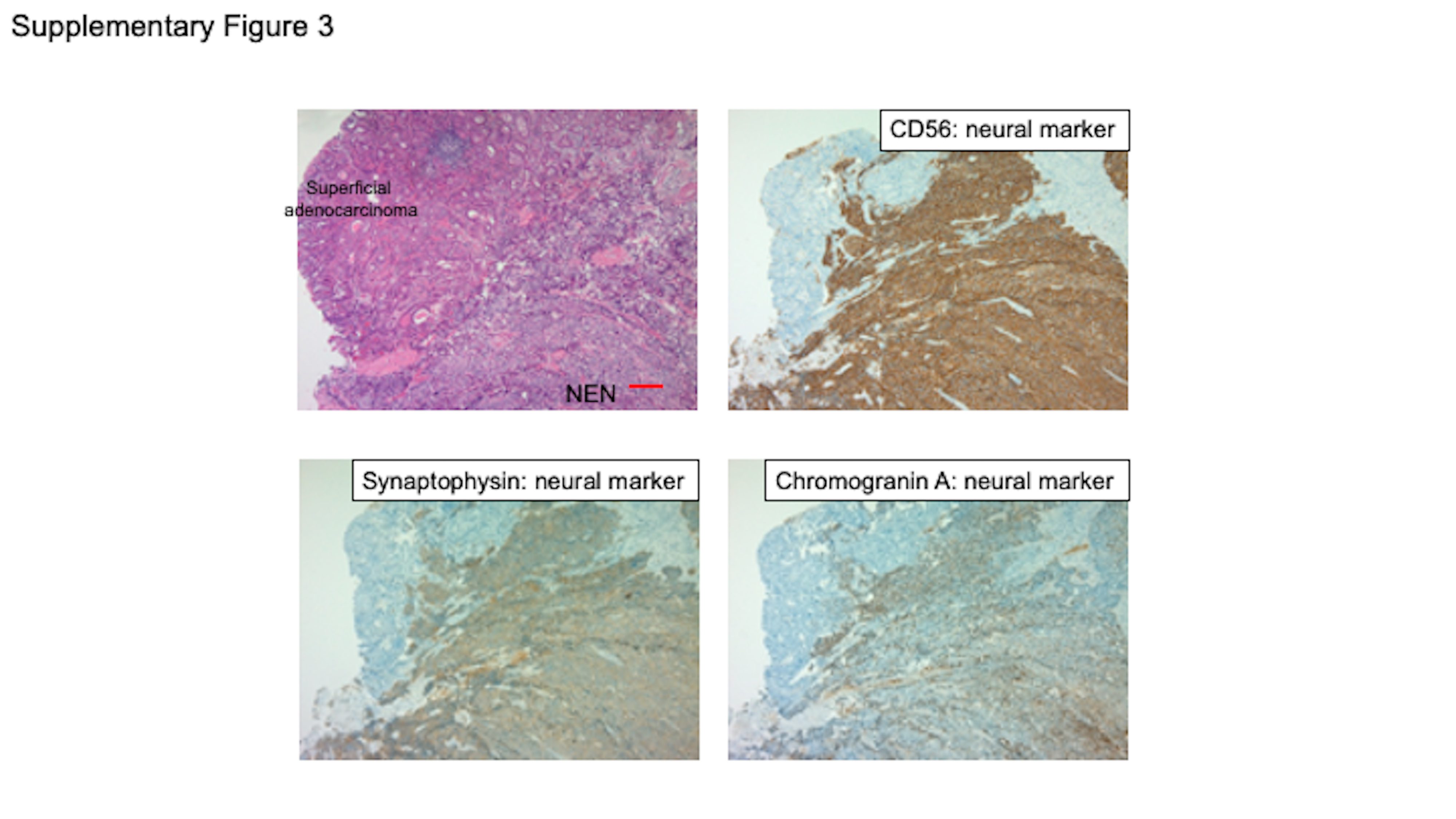

Supplement: Supplementary file 3 — FigS3 [file CAM4-11-983-s001.png]
